# GARP: A Key Target to Evaluate Tumor Immunosuppressive Microenvironment

**DOI:** 10.3390/biology10090836

**Published:** 2021-08-27

**Authors:** Alexanne Bouchard, Bertrand Collin, Carmen Garrido, Pierre-Simon Bellaye, Evelyne Kohli

**Affiliations:** 1 Centre George-François Leclerc, Service de Médecine Nucléaire, Plateforme d’Imagerie et de Radiothérapie Précliniques, 1 rue du Professeur Marion, 21079 Dijon, France; abouchard@cgfl.fr (A.B.); BCollin@cgfl.fr (B.C.); carmen.garrido-fleury@u-bourgogne.fr (C.G.); 2 UMR INSERM/uB/AGROSUP 1231, Labex LipSTIC, Faculty of Health Sciences, Université de Bourgogne Franche-Comté, 21079 Dijon, France; 3 Institut de Chimie Moléculaire de l’Université de Bourgogne, UMR CNRS/uB 6302, Université de Bourgogne Franche-Comté, 21079 Dijon, France; 4 CHU Dijon, 21079 Dijon, France

**Keywords:** GARP, TGF-β, cancer, biomarker, immunosuppression

## Abstract

**Simple Summary:**

Tumors are not only composed of cancer cells but also of various infiltrating cells constituting the tumor microenvironment (TME); all these cells produce growth factors which contribute to tumor progression and invasiveness. Among them, transforming growth factor-β1 (TGF-β1) has been shown to be a potent immunosuppressive cytokine favoring cell proliferation and invasion and to be associated with resistance to anticancer treatments. Glycoprotein-A repetition predominant (GARP) plays a critical role in the activation of TGF-β1 and has been shown to be expressed at the membrane of cancer cells and also of regulatory T cells and platelets in the TME. An increased GARP expression has been shown in a variety of cancers. The objective of this review is to highlight GARP’s expression and function in cancer and to evaluate its potential as a predictive and therapeutic follow-up biomarker that could be assessed, in real time, by molecular imaging.

**Abstract:**

Glycoprotein-A repetitions predominant (GARP) is the docking receptor for latent transforming growth factor (LTGF-β) and promotes its activation. In cancer, increased GARP expression has been found in many types of cancer. GARP is expressed by regulatory T cells and platelets in the tumor microenvironment (TME) and can be also expressed by tumor cells themselves. Thus, GARP can be widely present in tumors in which it plays a major role in the production of active TGF-β, contributing to immune evasion and cancer progression via the GARP-TGF-β pathway. The objective of this review is to highlight GARP expression and function in cancer and to evaluate the potential of membrane GARP as a predictive and therapeutic follow-up biomarker that could be assessed, in real time, by molecular imaging. Moreover, as GARP can be secreted, a focus will also be made on soluble GARP as a circulating biomarker.

## 1. Introduction

TGF-β, in particular the predominant isoform, TGF-β1, plays a major role in tumor progression due to its pleiotropic effects [1]. TGF-β is, in fact, a potent immunosuppressive cytokine, impacting antitumor immune responses [2] and it has many other protumor effects related to its role in epithelial–mesenchymal transition, cell proliferation, cell invasion and angiogenesis [3,4]. In addition, TGF-β has been reported to be associated with resistance to anticancer chemotherapies [5] as well as to immune checkpoint inhibitors [6]. TGF-β1 is thus considered a major immune checkpoint and a predictive and monitoring marker of response to treatments [4]. However, its use as a biomarker is made difficult by the existence of several inactive forms upstream of the biologically active TGF-β. Indeed, due to its potent effects, the production and secretion of the biologically active form of TGF-β is highly regulated. Glycoprotein-A repetition predominant (GARP) is considered the membrane docking receptor of latent-TGF-β (LTGF-β) [7,8] and it plays a critical role in the activation of TGF-β, as it allows the interaction of LTGF-β with integrins αvβ6 or αvβ8, a prerequisite step for the release of biologically active TGF-β [9,10]. Regulatory T cells (Tregs) are important producers of TGF-β, and GARP expression at their membrane is critical for their homeostasis and immunosuppressive function [8,11]. Besides Tregs, GARP has also been found at the surface of activated B cells [12], platelets [13,14], mesenchymal stem cells [15] and hepatic stellar cells [16]. In cancer, to date, GARP has been reported to be expressed by Tregs and platelets in the tumor microenvironment (TME, ref. [14,17]. In addition, it can be also expressed by tumor cells themselves [9,18]. Thus, GARP can be widely present in a tumor, both in tumor cells and in cells of the TME, where it plays a major role in the production of active TGF-β. Increased GARP expression has been shown in a variety of cancers, including breast [19], lung [17], melanoma [18], bone sarcoma [20], gastric [21], colon cancers [9,22], hepatocellular carcinoma [23], papillary thyroid carcinoma [24] and glioblastoma [25]. Moreover, GARP can be secreted as soluble GARP and found in the plasma [18,26,27].

The objective of this review is to highlight GARP’s expression and function in cancer and to evaluate the potential of membrane GARP as a predictive and therapeutic follow-up biomarker that could be assessed, in real time, by molecular imaging. Moreover, as GARP can be secreted, a focus will also be made on soluble GARP as a circulating biomarker.

## 2. GARP Expression, Structure and Function

### 2.1. GARP Expression

The human GARP gene was firstly isolated in the 11q13.5-11q14 chromosomal region in human breast cancer cells and defined as DI1S833E. The homologous sequence in mice is located on chromosome 7, region 7E-7F. The GARP gene contains two coding exons, encoding the signal peptide, as well as nine amino-acid residues and a leucin-rich repeat (LRR) containing a transmembrane protein, respectively [28]. The GARP gene is expressed in various tissues, including placenta, lung, kidney, heart, liver, skeletal muscle, pancreas and lymphoid tissues [29]. Additionally, the GARP gene has been detected in different cell types, such as Tregs and activated B lymphocytes, megakaryocytes and platelets, mesenchymal stromal cells (MSC), hepatic stellate cells and human umbilical vein endothelial cells [13,15]. Although the GARP gene is detected in many cell types, the expression of the GARP protein has been reported only at the membrane of Tregs [8,30], activated B cells [12], platelets [13,14,30], MSC [15] and hepatic stellate cells [16]. GARP expression has been shown to be regulated by microRNAs, especially miR-142-3p. Zhou et al. have demonstrated that miR-142-3p binds directly to the 3′ untranslated region of GARP and represses GARP protein expression [31]. GARP membrane expression has been shown to be dependent on the endoplasmic reticulum (ER) stress protein Gp96 [32] and, in Tregs, the absence of membrane GARP in Gp96 KO Tregs impairs Forkhead box protein 3 (FoxP3) expression and Tregs’ immunosuppressive functions. In cancer, an amplification of GARP gene, as well as GARP expression, has been found in tumor cells, particularly in invasive, metastatic or treatment-resistant tumors [33,34,35]. Moreover, single nucleotide polymorphisms (SNP) located in the noncoding regions of human GARP are associated with poor survival in patients with ovarian cancer [36].

### 2.2. GARP Structure

**Figure 1:**
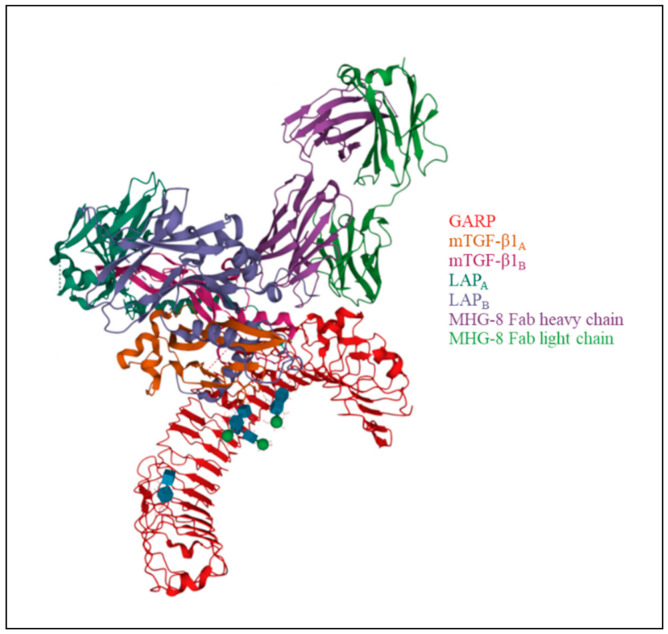
Structure of GARP in complex with latent TGF-β1 and MHG-8 Fab (PDB: 6GFF) [10,38]. LTGF-β1 contains two LAP (LAP_A_ and LAP_B_) and two mTGF-β1 molecules (mTGF-β1_A_ and mTGF-β1_B_).

GARP is a 72 kDa type I transmembrane protein consisting of 662 amino acids. Its structure consists of three regions: the extracellular domain, with leucine-rich repeats, accounting for about 70% of the protein; the hydrophobic transmembrane domain; and a cytoplasmic tail of 15 amino-acid residues [29,37]. The extracellular portion of GARP contains 20 LRR motifs, divided into two groups by a proline-rich region, and a C-terminal LRR. Two cysteines (positions 192 and 331, 7th and 12th LRR, respectively), are responsible for two disulfide bonds between GARP and LAP in LTGF-β [1,10] (Figure 1).

### 2.3. GARP Promotes the Activation of Biologically Active TGF-β

The 3D conformation of GARP suggests that it is a cell surface-acceptor molecule. Indeed, GARP was shown to be the docking receptor for LTGF-β [9,26,30,39,40,41] and was reported to promote the activation of LTGF-β through an integrin-dependent mechanism [9,10,41].

**Figure 2:**
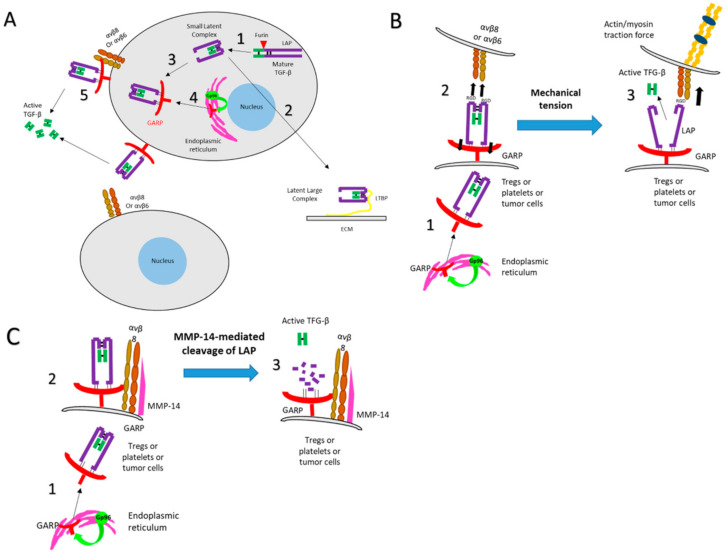
Multistep process leading to the release of biologically active TGF-β. (**A**) Latent TGF-β (LTGF-β). TGF-β is synthesized as a homodimeric precursor, cleaved by furin in the Golgi apparatus into LAP and the mature TGF-β, which remain noncovalently associated, as the latent TGF-β (LTGF-β, also called small latent complex) (**1**). This complex can associate with either the LTGF-β-binding protein (LTBP) into a large latent complex (LLC) that, in turn, associates with the extracellular matrix (ECM) (**2**), or GARP (**3**), which allows its membrane expression. GARP requires the ER chaperone Gp96 for its folding and surface expression (**4**). GARP orients the small latent complex (SLC) for binding and release by integrin αVβ8 or αVβ6 (**5**). (**B**) Activation of TGF-β through a protease-independent manner. After the SLC-GARP complex is expressed at the cell membrane with the aid of Gp96 (**1**), integrins bind to LTGF-β through the RGD motif on LAP (**2**). Cell contraction or mechanical tension induces a deformation of the surface LAP, mediating the release of the mature form of TGF-β (**3**). (**C**) Activation of TGF-β through a protease-dependent manner. After the SLC-GARP complex is expressed at the cell membrane with the aid of Gp96 (**1**), integrins recruit metalloproteinases (such as MMP-14) or serine proteases by an autocrine or paracrine pathway (**2**) which cleave LAP thus allowing TGF-β release (**3**).

TGF-β family members (β1, β2 and β3) are pleiotropic cytokines expressed by most cells. The three TGF-β isoforms are synthesized as a homodimeric precursor containing three distinct parts: the signal peptide, the latency associated peptide (LAP) and the mature TGF-β. After removal of the signal peptide, the precursor is further processed by a proteolytic cleavage at basic residues by furin, thus separating LAP from the mature peptide. LAP and the mature TGF-β remain noncovalently associated, to form the latent-TGF-β (LTGF-β), also called the small TGF-β complex, thus preventing the binding of active TGF-β to its receptor [7]. LTGF-β can then associate with either the LTGF-β-binding protein (LTBP) into a large latent complex (LLC) that in turn associates with the extracellular matrix (ECM) or GARP which allows its surface expression [8] (Figure 2A). Only LAP-free TGF-β is biologically active. The release of TGF-β from LAP represents a critical step for TGF-β function and signaling [9]; in addition to being the docking receptor for TGF-β at the cell membrane, GARP was reported to allow the activation of LTGF-β. Lienart et al. elegantly demonstrated that GARP can chaperone and orient LTGF-β for binding to integrin αVβ8 or αVβ6 [10,39] via an arginine-glycine-aspartate (RGD) motif. This interaction allows the further release of the biologically active TGF-β from LTGF-β through a protease-dependent—or independent—mechanism. In a protease-independent manner, integrin binds to LTGF-β, itself bound to GARP. Cell contraction or mechanical tension may therefore induce a deformation of the surface LAP, mediating the release of the mature form of TGF-β (Figure 2B). In a protease-dependent mechanism, integrin recruits a metalloproteinase or a serine protease by an autocrine or paracrine pathway that cleaves LAP and subsequently liberates TGF-β (Figure 2C). Tregs preferentially express the integrin β8 chain, so the activation of LAP-TGF-β from the LAP-TGF-β/GARP complex expressed on the surface of activated Tregs is mainly mediated by the integrin αVβ8 [42]. Based on the fact that no apparent pathology is associated with TGF-β deletion from Tregs, it has been proposed that the Treg-specific GARP may also absorb soluble LTGF-β produced by non-Tregs cells from the environment. Such a mechanism may not be restricted to Tregs [32].

TGF-β has many critical roles in numerous aspects of biological processes [1]. In cancer, TGF-β suppresses both the innate and adaptive immune systems, induces extracellular matrix deposition, invasion, loss of cellular adhesion, metastasis formation and angiogenesis [3,9]. An increased expression of GARP has been shown to increase the bioactivity of TGF-β and cause oncogenesis [1].

## 3. GARP in Cancer

GARP can be widely present in a tumor, both on tumor cells and on cells of the TME. By positively regulating TGF-β in the TME, GARP promotes oncogenesis. Moreover, GARP can be secreted (soluble GARP).

### 3.1. GARP and Cancer Cells

GARP is widely expressed by human cancer cells compared with normal epithelial cells [18]. An increase in GARP protein expression by cancer cells has been reported in many types of cancer: breast [19], lung [17], melanoma [18], bone sarcoma cancer [20], papillary thyroid cancer [24] and glioblastoma [25]. GARP may support cancer cell growth and dissemination by providing a reservoir of TGF-β that functions in the TME by suppressing the innate and adaptive immune responses, inducing extracellular matrix deposition, invasion, loss of cellular adhesion, metastasis formation and angiogenesis. Metelli et al. showed that overexpression of GARP in the 4T1 murine mammary carcinoma cell line increased TGF-β activation, tumor growth, metastasis and immunosuppression [9]. Similarly, Hahn et al. showed that GARP is expressed on human melanoma cells and plays a critical role in setting up an immunosuppressive TME [18]. GARP is also expressed on bone sarcoma cells lines. Knocking-down GARP in these cell lines decreased their proliferation and induced apoptosis whereas overexpression of GARP increased their growth in vitro and in vivo as well as their resistance to chemotherapy and radiotherapy [20]. Furthermore, GARP itself has a transformation potential. Indeed, it can render normal mammary gland epithelial cells tumorigenic [9]. In fact, a variant of the NMuMG* cell line, which expressed a high level of endogenous GARP, has been recently described as capable of forming tumors in mice by Metelli et al. whereas silencing of GARP alone in these cells significantly attenuated their growth in vivo [43]. This may suggest that increased expression of GARP could lead to cancer in this model [9].

### 3.2. GARP and Cells of the TME

#### 3.2.1. Tregs

FoxP3 Tregs, a highly immunosuppressive subset of CD4^+^ T cells, plays a major role in immune homeostasis by controlling the immune responses. GARP is found to be highly expressed on the surface of activated Tregs and to maintain their regulatory functions [44]. Infiltration of Tregs expressing GARP in the TME is associated with poor prognosis in various types of cancer, including melanoma [18], lung [17], colon [22] and gastric [21] cancers. Indeed, Tregs have been shown to inhibit antitumor immune responses, thus enhancing tumor progression [17,18,21,22].

Of note, Jin et al. have shown that lung cancer cells could induce GARP expression on Tregs Lung cancer cells could induce GARP expression on Tregs by secreting some cytokines, which need to be identified. However, this was cell line-dependent, A549 and H520 cells inducing high GARP expression on Tregs conversely to H460, LTEP-A-2, and GLC-82 cells [17].

GARP favors a regulatory network between Tregs and their targets. Indeed, GARP is involved in the function of Tregs via the secretion of biologically active TGF-β which can act in a paracrine manner and play a critical role on natural killer (NK) cells, as well as CD4^+^ and CD8^+^ T cells. TGF-β from Tregs is also able to act in an autocrine manner, maintaining their function and homeostasis [45]. Indeed, the binding of TGF-β to its receptor activates the Smad pathway that induces the expression of FoxP3 [46].

The link between GARP and FoxP3 remains controversial. On the one hand, FoxP3 and GARP expression appears independent as the expression of GARP is unaffected in FoxP3 knockdown Tregs and silencing GARP only attenuates Tregs suppressive activity but does not affect the expression of FoxP3 [30,47]. On the other hand, Probst-Kepper et al. [48] suggest an interdependence via a positive feedback loop between GARP and Foxp3. By downregulating GARP or Foxp3 in a CD4^+^ CD25^hi^-derived Tregs cell line using shRNA, they showed that GARP-specific shRNA decreased GARP and FoxP3 expression. This was associated with an impaired induction of CD83 and CD27, both known to regulate Foxp3 [49,50], suggesting an interrelated network of FoxP3-regulating systems in Tregs. These results are in accordance with other studies showing that (i) the silencing of FoxP3 in human Tregs reduced surface GARP expression; (ii) downregulation of GARP, in Tregs, significantly impaired their suppressor function, together with downregulation of FoxP3; and (iii) GARP overexpression in naïve T cells induced the expression of FoxP3 [11,48,51].

#### 3.2.2. Platelets

Platelets have been found to constitutively express GARP with increased expression upon platelet activation [14]. Besides their essential role in hemostasis [52], platelets are involved in angiogenesis, wound healing and immunomodulation [53,54,55]. In cancer, they have been shown to promote tumor invasion [56,57,58,59] and cancer-associated thrombocytosis is an independent poor prognosis factor in multiple cancer types [60,61,62]. Tumors continually activate the coagulation pathways, which leads to thrombin formation and chronic platelet activation. Rachidi et al. have isolated T cell-specific immunomodulators from platelets and identified TGF-β as the most important to suppress CD4^+^ and CD8^+^ T cells [14]. Extracellular TGF-comes from the release of pre-stored TGF-β in the cytoplasmic granules or via the ability of surface GARP on platelets to bind LTGF-β from nonplatelet sources. Rachidi et al. have demonstrated that platelet-intrinsic GARP, which is constitutively expressed, plays the most dominant role in activating TGF-β between these two options and thus likely contributes to the immunosuppressive molecular hallmarks in the TME [14]. TGF-β secreted from activated platelets enhances EMT in cancer cells [58] and plays an important role in metastasis formation [63]. It inhibits NK cells antitumor activity by downregulating NK group 2 member D (NKG2D) on NK cells [64]. In addition, Hu et al. have shown that TGF-β secreted from activated platelets promote tumor growth ovarian cancer by increasing the proliferation of cancer cells [63]. Of interest, platelets support the in situ activation of prothrombin to active thrombin, which can cleave LTGB-β and release the mature TGF-β (see paragraph “GARP promotes the secretion of biologically active TGF-β”). To prove the connection between thrombin and GARP and the release of TGF-β mature, platelets from wild type (WT) and from platelet-specific GARP knockout mice have been isolated and activated in the presence or absence of thrombin by Metelli et al. [65]. Thrombin activation enhanced mature TGF-β release from WT but not from GARP KO platelets. Thus, GARP is necessary for platelet TGF-β release. Finally, the authors showed that inhibition of thrombin using dabigatran etexilate (a competitive and reversible direct thrombin inhibitor approved for venous thromboembolic events) reduced the GARP-dependent production of TGF-β from platelets, blocked TGF-β deposition in both the tumor bed and the stroma of colon carcinoma [65] and allowed the increase of CD8 T cells [66], NK cells [64] and neutrophils [67] in the TME [65].

Altogether, these results suggest that coagulation and platelet activation may contribute to immune evasion and cancer progression via the GARP- TGF-β pathway.

#### 3.2.3. Other Cells

To our knowledge, GARP expression by other known GARP-expressing cells that may be present in the TME has not yet been reported in cancer. Among them, B cells, which express GARP when activated by TLR ligands such as TLR4, TLR7 and TLR9, have been only explored in the context of autoimmune disease [12] and hepatic stellate cells, for which GARP is required to anchor and activate LTGF-β, are currently being explored in liver fibrosis [16].

### 3.3. Soluble GARP

Ectodomain shedding is a proteolytic mechanism by which transmembrane molecules are converted into a soluble form. This allows the cell to rapidly adopt a distinct surface phenotype and to generate soluble mediators that can act on other cells. GARP was thought to require membrane anchoring to exert its regulatory function, because its soluble LTGF-β associated form was found to be unable to support αVβ6- or αVβ8-mediated TGF-β activation [68]. A shedding process for GARP was first proposed by Roubin et al. in 1996 [29]. The authors hypothesized that the hydrophobic leader sequence domain might be the signal peptide for targeting the protein to the secretory pathway [28]. Soluble GARP (sGARP) was further found in human plasma in cancer [1,69].

#### 3.3.1. Modulation of T-Cell Function by Soluble GARP

Hahn et al. have shown a tolerogenic effect of sGARP by generating a soluble recombinant human GARP protein and analyzing its impact on the differentiation and activation of human CD4^+^ T cells. They demonstrated that sGARP induced FoxP3 expression, decreased proliferation and suppressed IL-2 and IFN-γ production in naïve CD4^+^ T cells but not in differentiated CD4^+^ T cells, resulting in differentiation of naïve T cells into induced Tregs. FoxP3 induction and repression of cytokines by sGARP was abrogated after blocking the TGF-β receptor [26]. These findings can be interpreted as indirect evidence for an interaction of sGARP and soluble LTGF-β in the extracellular space. Fridrich et al. have shown that sGARP could bind free LTGF-β non-covalently and enhance its activation. All of these findings support the hypothesis that sGARP could bind to exogenous LTGF-β and improve its activation [39]. Interestingly, sGARP had similar effects as biologically active TGF-β on FoxP3 induction and cytokine (IL-2 and IFN-γ) repression. The blockade of TGF-β signaling equally diminished sGARP and TGF-β effects on FoxP3 regulation and IL-2 production. These results have demonstrated that the effects of s-GARP on T cell modulation are, at least, in part associated with TGF-β signaling. Moreover, sGARP has been shown to induce phosphorylation of TGF-βR downstream targets such as Smad2/3 [26].

In cancer, the role of sGARP has been analyzed in vitro on CD8^+^ effector T lymphocytes (CTLs) stimulated with allogeneic melanoma cells. The results have shown a reduction in CTLs as well as a suppression in the frequency of granzyme expressing cells and in the total quantity of granzyme B [18].

#### 3.3.2. sGARP Influences the Polarization of Macrophages

Hahn et al. have reported that adding sGARP to the culture of M0 macrophages prevented the expression of M1 markers CD80 and CD16, but increased the M2 marker CD206. In addition, cytokine production (IL-8, IL-6, IL-10, and TNFα) of sGARP-treated macrophages showed similarities with a M0/M2 macrophage profile, known to have tumor promoting properties. On the other hand, no effect of sGARP was observed after treatment of M1 and M2 differentiated macrophages [18]. Altogether these data demonstrate that sGARP skews macrophages to an alternatively activated phenotype and could be involved at least in part in M2 macrophage polarization. Tumor-associated macrophages (TAM) generally express an M2-like phenotype and play an essential role in tumor progression by stimulating angiogenesis, tumor cell invasion and metastasis, and suppressing anti-tumor immunity [70]. Accordingly, a high TAM number is correlated with a poor prognosis in patients with various cancers [71].

Consequently, sGARP could be considered a new soluble factor favoring M2 polarization, which also supports an immunosuppressive TME. Of note, we have shown that primary human M2 macrophages do not express GARP at their membranes, although they produce TGF-β [72] and, to our knowledge, M2-like macrophages in the TME have not been reported to express GARP.

## 4. GARP as a Therapeutic Target in Cancer

The role of GARP in the production of the active form of TGF-β and in Tregs homeostasis makes it an interesting target in some cancers [41]. The advantage of GARP over FoxP3 is its membrane localization allowing the use of therapeutic antibodies. Cuende et al. [73] have generated two anti-GARP monoclonal antibodies (mAbs) that block the production of active TGF-β by human Tregs. These antibodies recognize a conformational epitope within the GARP-TGF-β interaction site. The blocking anti-GARP mAbs inhibited human Tregs both in vitro and in vivo. More recently, one of these mAbs was used by De Streel et al. [74] to overcome resistance to anti-PD-1 therapy in tumor-bearing mice. These results make blocking anti-GARP-TGF-β interaction using mAbs an interesting approach to treat patients with cancer resistant to currently available immunotherapies (like immune checkpoint inhibitors). A phase I trial was recently initiated to test such antibodies in the clinics (ClinicalTrials.gov: NCT03821935). These anti-GARP antibodies, blocking the GARP-TGF-β interaction, could have advantage in cancer by transiently inhibiting the immunosuppressive function of Tregs without physically eliminating them, as this is the case for CTLA-4 antibodies [74,75]. In addition, these anti-GARP antibodies only block TGF-β produced by GARP-expressing cells, whereas anti-TGF-β neutralizes activity of all TGF-β, regardless of their cellular source, reducing side effects.

Most interestingly, Xing et al. recently demonstrated that GARP was also found in extracellular vesicules (exosomes) from MSC [76]. The knock-down of GARP in MSC-exosomes prevented proliferation, migration and invasion of human colon cancer cells compared with MSC-exosomes containing GARP. The authors identified that the lack of GARP in MSC-exosomes inhibited IL-6 signaling and JAK1/STAT3 pathway revealing the blockade of GARP in exosomes as a potential strategy for cancer therapy [76].

In addition, the specific deletion of GARP in platelets has been shown to inhibit TGF-β signaling, thus promoting anti-tumor immunity in various cancer types [14]. These findings highlight a novel therapeutic strategy in cancer based on the combination of GARP inhibition with platelet inhibitors.

Finally, compared to FoxP3, GARP is expressed by a greater diversity of cells contributing to the immunosuppressive environment including tumor cells themselves and platelets that are now recognized to play a major role in tumor progression via the production of biologically active TGF-β [65].

## 5. GARP as a Biomarker in Cancer: Perspectives

The definition of new biomarkers reflecting the immunosuppressive potential of a tumor, both for prognosis and treatment options, has become essential to personalize disease management. Regarding its expression on tumor cells and on immunosuppressive cells of the TME, as well as its function via the TGF-β activation, GARP may represent an interesting biomarker. GARP has been reported to be overexpressed in a variety of cancers, among them, breast [19], lung [17], melanoma [18], bone sarcoma [20], gastric [21], colon cancers [9,22], hepatocellular carcinoma [23], papillary thyroid carcinoma [24] and glioblastoma [25]. As GARP can also be secreted in the plasma, an easy approach should be to quantify GARP in the serum. To our knowledge, the quantification of GARP in the serum has not been reported in the literature. GARP expression was quantified by flow cytometry on platelets from peripheral blood (PB) of patients with melanoma. Independent of their tumor stage, melanoma patients have shown a significant upregulation of GARP on the platelet surface, compared with healthy donors. Nevertheless, there was no difference in GARP expression between early- and late-stage melanoma patients [62]. GARP expression was also quantified by flow cytometry in Tregs from PB by Jin et al., but no difference was shown between subjects with lung cancer and healthy subjects [17]. Interestingly, the proportion of GARP^+/^FoxP3^+^ Tregs is elevated in hepatocellular carcinoma and GARP is significantly upregulated in FoxP3^+^ Tregs in these patients [23]. In tumors, GARP expression-levels on Tregs are significantly increased, compared with that in PB in lung cancer. Of note, the authors found that GARP was more expressed on Tregs from tumors of patients at stages I and II, compared with patients at stages III and IV. They propose that GARP might be an early prognostic biomarker. Concerning the global expression of GARP in tumor biopsies, it has been investigated by Zhang et al. in papillary thyroid cancer [24]. A significant increase in the expression of GARP was found in papillary thyroid cancer compared with benign thyroid diseases, including nodular goiter and adenoma, however, no significant association of GARP expression with the clinical stages of patients was observed. Carillo-Galvez et al. have also analyzed GARP expression in tumor biopsies of human bone sarcoma by immunochemistry and found that high expression of GARP was correlated with worse overall survival [20]. Although none of the clinical–pathological variables analyzed showed a significant correlation with levels of GARP expression, a tendency was observed for a higher grade and mitotic count-scoring in tumors showing high expression of GARP. GARP expression could be included in a multivariate survival analysis. A tendency was observed, correlating the expression of GARP and poor prognosis, however, before it can be made an independent prognostic and predictive marker in bone sarcoma management, further correlations between GARP expression and outcome need to be performed on additional bone sarcoma samples.

In conclusion, the expression of GARP has been shown to be increased in multiple cancers and could be considered a biomarker. However, the correlation between the expression of GARP and the different stages of these cancers remains to be demonstrated to confirm its use as a prognostic biomarker. Comparisons between early and advanced stages of cancer may be difficult without treatment information. Advanced stages are most often under treatment, which could impact GARP expression. Indeed, Kai Li et al. have shown that neoadjuvant chemotherapy decreased the infiltration of GARP^+^ Tregs in intratumoral gastric cancer [21] and suggested that GARP could, therefore, represent a marker for therapeutic monitoring and response to treatment. In addition, Carrillo-Galves et al. have shown that the implantation of tumor cells with enhanced GARP expression led to increased tumor growth, as well as resistance to chemotherapy and radiotherapy. Thus, GARP might also serve as a predictive marker of resistance to treatment [20]. In this context, in vivo molecular imaging (e.g., positron emission tomography, PET) targeting GARP could then represent an interesting tool. However, to our knowledge, there is currently no data on GARP imaging in cancer. This approach could allow in vivo phenotyping and monitoring of the tumor to determine its aggressiveness, treatment options and responses, in a noninvasive, rapid and personalized manner.

## 6. Conclusions

GARP is the docking receptor for LTGF-β and promotes its activation. In cancer, increased GARP expression has been shown in many cancers of bad prognosis, both in tumor cells and in cells of the TME, where it plays a major role in the production of active TGF-β, thus contributing to the immunosuppressive environment. Regarding its expression as well as its function via TGF-β activation, GARP may represent an interesting biomarker for prognosis and therapeutic follow-up. In vivo molecular imaging, such as PET and targeting GARP, could represent an interesting, personalized approach to further investigate GARP potential as such a biomarker in cancer.

## Data Availability

Not applicable.
